# Research based on existing clinical data and biospecimens: a systematic study of patients’ opinions

**DOI:** 10.1186/s12910-022-00799-4

**Published:** 2022-06-16

**Authors:** Hilde Eikemo, Linda Tømmerdal Roten, Arne Einar Vaaler

**Affiliations:** 1 Regional Committee for Medical and Health Related Research Ethics Mid Norway, Faculty of Medicine and Health Sciences, Norwegian University of Science and Technology (NTNU), REK Midt V/Hilde Eikemo, Postboks 8900, 7491 Torgarden, Trondheim, Norway; 2 Østmarka Department of Psychiatry, St. Olav University Hospital, Trondheim, Norway; 3 Department of Mental Health, Faculty of Medicine and Health Sciences, Norwegian University of Science and Technology, Trondheim, Norway

**Keywords:** Patient opinions, Active consent, Passive consent, Secondary research, Opt-in, Opt-out

## Abstract

**Background:**

The aim of the present survey was to investigate newly discharged hospital patients’ opinions on secondary use of their hospital data and biospecimens within the context of health research in general and, more specifically, on genetic research, data sharing across borders and cooperation with the health industry.

**Methods:**

A paper questionnaire was sent to 1049 consecutive newly discharged hospital patients.

**Results:**

The vast majority of the respondents preferred to be informed (passive consent) or to receive no notification at all for secondary research on their health data and biospecimens (88% and 91% for data and biospecimens respectively). The rest wanted to be asked for active consent. The same trend applied for the other aspects also. 81% of respondents were positive towards genetic research without active consent. 95% were positive towards cooperating with the health industry, and 90% were positive towards data sharing.

**Conclusions:**

These results suggest that hospital patients generally are very positive to secondary research and support the concept of opting out rather than opting in.

**Supplementary Information:**

The online version contains supplementary material available at 10.1186/s12910-022-00799-4.

## Background

Health data and biospecimens from ordinary clinical practice are valuable sources for secondary research due to their clinical relevance. It is possible to analyze outcomes for “real-life” patients in ordinary clinical settings [1]. More access to detailed clinical data and the possibility for follow-up data from public health registries will increase the value of this information. However, identification of individual patients is necessary for data linkage. Thus, individual research participants need to be identified from health records. This may require consent [2].

Consent has been regarded as one of the pillars of research ethics for decades. It should be voluntary, informed, and explicit. Informed consent is justified by the principle of autonomy.

Secondary research can be defined as research using only already collected data and biospecimens, which may stem from various sources. In the following text we refer to secondary research that makes use of clinical data and biospecimens for research purposes. Such reuse is generally considered to pose minimal risk to participants with appropriate handling of private information. Scholars debate whether active consent to secondary research is necessary and, if required, whether it should be justified in terms of courtesy or risk [3]. Many countries have developed options for proportional review of such studies. Still, legislation in different countries shows huge variation when it comes to regulating secondary research using data from hospital records and diagnostic biobanks [4]. In Norway, where the present study was carried out, active consent is the main rule for both interventional and non-interventional research. Exemption rules exist, but are only used if strict criteria are met, such as minimal risk to the research participant, significant societal benefit, and that it is difficult to obtain active consent. For the use of biospecimens in health research, the research participants must also receive information about such use in beforehand and be given the opportunity to make a reservation from such use [5].

Multiple models for informed consent have been developed [6]. The models of consent may be active, requiring that participants actively provide their consent (“opt-in”), or passive where patients are informed about the project and included unless they express their desire to be excluded (“opt-out”). The active consent model is embedded in several countries’ health research legislation. The appropriateness of passive consent has long been debated and is often considered to have both a low ethical and legal status [7].

“Broad consent” can be defined as “consent for an unspecified range of future research subject to a few content and/or process restrictions” [2]. If certain conditions are fulfilled, this model has general support from patients, researchers, and ethicists [2, 6]. One condition is that the secondary research should be supervised by external and independent bodies monitoring procedural correctness [6].

Patients may or may not be asked for their consent to secondary research at the time of examination and treatment. Consent forms, when available, often have varying degrees of specificity [8]. This is a challenge for institutional review boards (IRBs)/research ethics committees (ECs)/research ethics boards (REBs) and researchers when considering secondary research outside the scope of the original data subjects’ consent [1].

There are a limited number of surveys exploring hospital patients’ opinions regarding secondary research using their clinical, hospital-derived data. However, similar surveys on this topic have been investigated in general populations and in biobank research [9] and reviewed and debated by others [10–13]. Different methods of patient selection have been used, including “patients being enrolled from a convenience sample at clinic appointments” [14]. Other researchers have used structured or semi-structured telephone interviews. The studies generally have shortcomings, making it difficult to draw firm conclusions. For instance, participants are seldom recruited in a systematic manner. This leads to uncertainty regarding the representativeness of the samples, the number of drop-outs, the clinical condition of patients, the seriousness of the illnesses, whether participants are inpatients or outpatients, and time since treatment. Limited knowledge exists about hospital patients’ views on future linkage of data for secondary research, for instance linkage between medical records, their specific phenotype, and public health registries.

A number of surveys have assessed views on the use of stored biospecimens in secondary research in different populations (reviewed by Domaradzki et al. [10]). Chen and colleagues found that most participants “authorize the unlimited future use of their biological samples when given the opportunity to do so” [6]. However, this is an example of a more positive attitude. Generally, respondents want to decide whether their biospecimen can be used [2, 8]. Schwartz et al. (2001) found that more than half of the respondents in their study believe researchers should obtain written informed consent before doing secondary research [15]. Most surveys assessing public attitudes towards storing biospecimens for prospective secondary research have addressed different populations than “real-world” hospital patients. Population studies have similar shortcomings [16]. We are not aware of any study that addresses hospital patients’ opinions on secondary research on hospital data and biospecimens supplemented with alignment to public health registries.

The aim of the present survey was to systematically assess the opinions of recently discharged hospital patients regarding various aspects of secondary research on their hospital data and biospecimens.

## Methods

### Population and recruitment

During the time period of May 2018 to February 2019, 1049 patients were recruited in a systematic manner from seven different departments at St. Olav’s Hospital, Trondheim University Hospital, Mid Norway. These were the Women’s Clinic, the Clinic of Medicine, the Cancer Clinic, the Clinic of Surgery, the Department of Endocrinology, the Clinic of Thoracic and Occupational Medicine, and two district psychiatric centers. The seven departments were chosen to ensure a mix of both sexes, all ages (> 18 years), various degrees of seriousness of illnesses, and both somatic and psychiatric disorders. Sample size was determined based on feasibility. The patients were a mix of inpatients and outpatients. Norwegian inpatient somatic and psychiatric services are based on catchment areas, primarily publicly funded, and open to anyone. All patients from the catchment area in need of acute and emergency services, and most of the elective patients in need of inpatient services, are admitted to the hospital. In addition, St. Olav’s Hospital has an extensive outpatient activity serving every specialty in clinical medicine.

The staff of each clinic/department sent an envelope to the home address of 150 previously hospitalized patients including all discharged patients starting three months prior and going backwards on the patient list. The staff excluded patients in cases where the lack of capacity to consent to health interventions was documented in the health records.

The envelopes sent to the patients contained an invitation to take part in the survey (see Additional file 1), the questionnaire (see Additional file 2) and a postage paid envelope to be returned to the research group. The patients consented to take part in the survey by filling out and returning the questionnaire. They were informed that the researchers would only receive deidentified data.

In order to enhance the recruitment, participants who returned the questionnaire were entered in a drawing to win an iPad. To be able to identify a winner, each questionnaire was marked with a unique number. The identity of the patient receiving the specific number was known only to the staff at the hospital departments. The staff noted the sex and age of all invited patients.

### The questionnaire

The 12-item questionnaire had two to four response alternatives for every item (see Additional file 2). It was initially developed by the authors and subsequently refined through an extensive expert review process including the director of research at the hospital, medical specialists from the participating clinical departments, members of the local research ethics committee, former patients, laypersons and the hospital’s service use advisory group. The text was in Norwegian.

The single items included topics such as the preferred level of consent for secondary research on already collected health information, biospecimens, and genetic data, as well as data sharing across national borders and cooperation with the health industry. The questions did not specify the state (i.e. identifiable vs. de-identifiable vs. anonymized) of the hospital data and biospecimens.

For decades, Norway has kept national health registries (e.g. the Cancer Registry, the Education Registry, the Cause of Death Registry) that collect information on all residents. Individuals are identified with a unique, eleven-digit number. Information from a hospital stay can be linked with retrospective and/or prospective data using the registries. A question regarding such linkage was also included.

A limited number of demographic variables (age group, sex and hospital department) were added to the questionnaire.

### Statistical analyses

The differences in the characteristics of respondents and non-respondents were analyzed in IBM SPSS Statistics 26 using crosstabs and Pearson Chi-square tests with a significance level of 5%. P-values were computed by using Pearson Chi-square tests in a 2 × 2, 2 × 5 and 2 × 7 contingency table comparing respondents and non-respondents for the variables sex, age, and department, respectively.

## Results

### Response rate

Out of 1049 requests for participation, fourteen were returned unopened. The total response rate was 41% (424 out of 1035 requests). According to national legislation, information about sex, age and department can be collected without active consent in order to compare respondents and non-respondents. However, six of the non-respondents actively refrained from sharing even this limited data for scientific purposes. For one of the non-respondents we only have information about which clinic the questionnaire was sent from.

### Respondents and non-respondents

Age, sex and department of respondents and non-respondents is presented in Table 1.

**Table 1:** Characteristics of respondents and non-respondents

	Respondents (%)	Non-respondents (%)
Sex		
Female	265 (62.5)	365 (60.3)
Male	159 (37.5)	239 (39.5)
Missing		1 (0.2)
Total	424 (100)	605 (100)
Age (years)		
18–29	61(14.4)^a^	154 (25.5)
30–39	68 (16.0)	109 (18.0)
40–49	38 (9.0)	84 (13.9)
50–69	153 (36.1)	148 (24.5)
70+	100 (23.6)^b^	65 (10.7)
Missing	4 (0.9)	45 (7.4)
Total	424 (100)	605 (100)
Department		
Women’s clinic	59 (13.9)	91 (15.0)
Cancer clinic	85 (20.0)^c^	65 (10.7)
Clinic of medicine	58 (13.7)	87 (14.4)
Clinic of surgery	63 (14.9)	83 (13.7)
Department of endocrinology	66 (15.6)	81 (13.4)
Clinic of thoracic and occupational medicine	59 (13.9)	85 (14.0)
Tiller and Nidaros DPS	34 (8.0)^d^	113 (18.7)
Missing		
Total	424 (100)	605 (100)

Data presented as number/count (percentage). *P* values are computed by using Pearson Chi-square test in a 2 × 2, 2 × 5 and 2 × 7 contingency table (crosstabs) comparing responders and non-responders for the variables sex, age and department respectively

^a,b^Significantly fewer discharged patients in the age group 18–29 years participated, whereas in the age group 70 years and older (70+) significantly more participated (χ^2^ = 55.714, *p* value = 2.3*10^–11^)

^c,d^Significantly fewer patients at the district psychiatric centers participated, whereas significantly more patients at the Cancer clinic participated (χ^2^ = 35.943, *p* value = 0.000003)

Chi square analyses revealed no statistically significant differences between respondents and non-respondents in terms of sex, but women made up a majority of those that were asked for participation. Significantly fewer discharged patients in the age group 18–29 years answered the questionnaire, whereas in the age group 70 years and older (70 +) significantly more answered the questionnaire. Significantly fewer patients at the district psychiatric centers answered the questionnaire, whereas significantly more patients at the cancer clinic answered the questionnaire.

### The respondents’ opinions

**Fig. 1:**
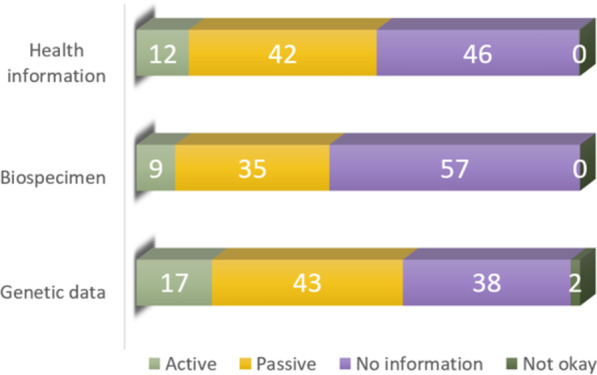
The respondents’ opinions on secondary research in their health information, their biospecimen and the genetic data derived from it (presented as percent). We asked if secondary research in health information, biospecimen and genetic data was okay (1) with active consent ("opt-in"), (2) with passive consent ("opt-out"), (3) without information or (4) not okay

We asked the newly discharged patients about their opinions regarding the appropriate level of consent for secondary research on already collected health information, biospecimens, and genetic data, and sharing of health information and biospecimen across national borders. Figure 1 shows their opinions on level of consent for secondary research on health information, biospecimens, and genetic data (questions 1–3). 12% of the respondents preferred an active consent model (“opt-in”), 42% preferred passive consent (“opt-out”), and 46% did not think it necessary to consent or receive any information about their health information being used in secondary research. For the future use of biospecimens, 9% of the respondents wanted to consent actively, 35% wanted to give passive consent, and 57% did not think it necessary to consent or receive any information about their biospecimens being used. When it comes to secondary research on genetic data, 17% of the respondents preferred an active consent model, 43% preferred passive consent, and 38% did not think it necessary to consent or receive any information about their genetic data being used at all. Only 2% of the respondents did not want their genetic data to be used in secondary research.

Regarding the linkage of hospital data to other sources of information such as national registries (question 5), 10% of the respondents wanted an active consent model, 39% wanted a passive consent model, and 50% did not think it necessary to consent or receive any information about their health information being linked to other sources. Only 1% of the respondents did not want their hospital data to be linked to other sources.

**Fig. 2:**
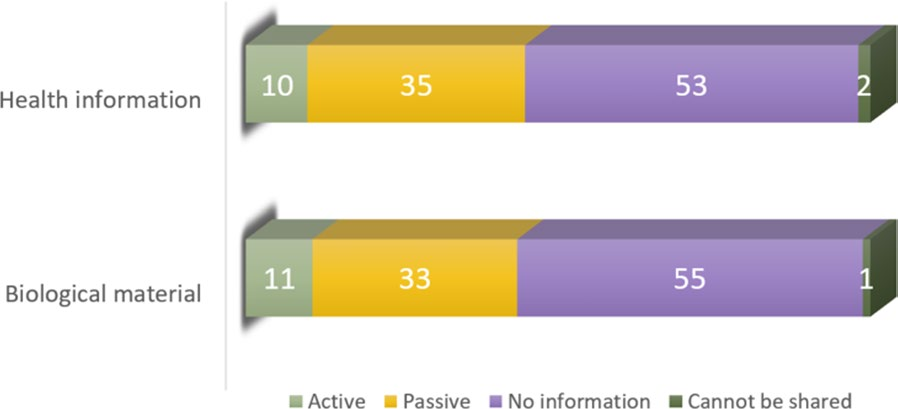
The respondents’ opinions on data sharing across national borders (presented in percent). We asked if their health information and biospecimen could be shared "around the world" (1) with active consent, (2) with passive consent, (3) without information or (4) cannot be shared

Figure 2 shows the respondents’ opinions on level of consent regarding sharing health information and biospecimen across national borders (question 9 and 10). 10% of the respondents wanted an active consent model, 35% wanted a passive consent model, and 53% did not think it necessary to consent or receive any information about their health information being shared.

Whereas 2% of the respondents did not want their health information to be shared, 11% of the respondents wanted an active consent model, 33% wanted a passive consent model, and 55% did not think it necessary to consent or receive any information about their biospecimens being shared. 1% of the respondents did not want their biospecimens to be shared.

**Fig. 3:**
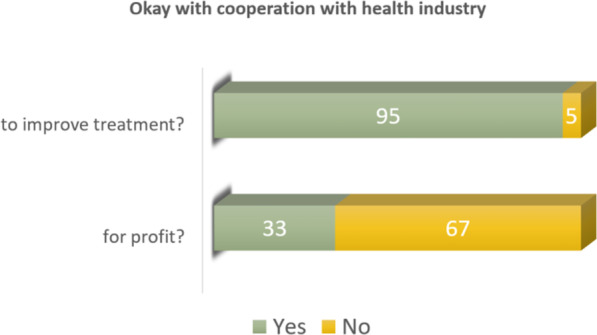
The respondents’ opinions on cooperation with the health industry in two different contexts (to improve treatment, or for profit)

We also asked the newly discharged patients about their opinions on researchers’ cooperation with the health industry in two different contexts (questions 6 and 7). The two contexts are (1) cooperation to improve treatment and (2) cooperation for profit. Figure 3 shows that 95% and 5% of the respondents, respectively, are okay or not okay with their biospecimens being used in research cooperation with the health industry with the aim of improving treatment. When the aim of the research cooperation is profit, 33% and 67% respectively are okay or not okay with their biospecimens being used.

The respondents were asked if they prefer a broad consent or specific consent model (question 11). The majority of the respondents, 62.5%, answered that they preferred a broad consent model, whereas 37.5% preferred a specific consent model.

**Fig. 4:**
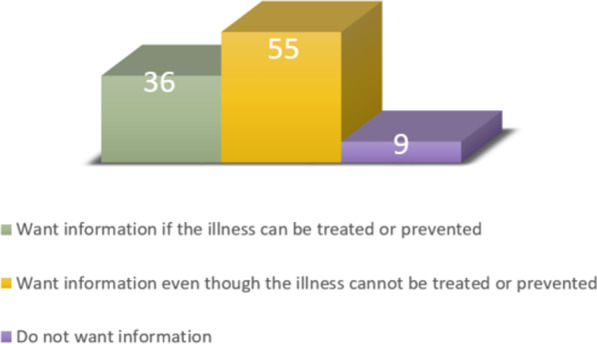
The respondents’ petition for information about results of genetic testing (presented as percent)

The newly discharged patients were also asked about their petition for information about results of genetic testing (question 4). Figure 4 shows that the majority of the respondents, 55%, wanted information about the results of genetic testing even when an illness cannot be treated or prevented. 36% of the respondents wanted information about the results only if the illness can be treated or prevented, and 9% did not want any information about the results.

Additional file 3 summarizes the respondents’ answers as frequencies and percentages for all twelve questions in the questionnaire.

## Discussion

### A positive attitude towards secondary research

In general, the respondents had a very positive attitude towards secondary research. Close to 90% preferred either passively consenting (“opt-out”) to the use of their health data or not being informed at all. They were even more positive towards the use of biospecimens. Only one patient (0.2%) declined to let their health information be used in secondary research. No patients objected to the use of their biospecimens.

Chen et al. (2005) reviewed consent forms from 61 previous studies involving questions on stored biospecimens [6]. The study populations were mixed including patients, family members, and healthy volunteers. They found that close to 90% “authorized future research on any medical condition.” However, their review is somewhat limited by the lack of information on drop-out rates in the different included studies [6]. Other surveys have similar shortcomings, but results frequently indicate more reluctance to secondary research on stored biospecimens [2, 14]. In a study from 2008, Hull et al. found that knowing what research was performed was important to 72% and 81% of respondents, depending on whether the specimens were anonymous or not [14]. The results from Chen et al. (2005) are supported by the present study indicating that patients are positive towards secondary research both on their health data and on biospecimens. In contrast, the legislation in many countries is based on a moderate protectionist approach saying that humans should be protected against undue risk in research [17]. There may seem to be a disharmony between legislation and patients’ perspective.

Data and biospecimens from routine healthcare have the potential to transform the healthcare services. Access to data from examinations, treatments, and “real-life” patient outcomes in ordinary clinical settings is crucial, not only for quality control but also for the further development of personalized medicine and artificial intelligence to improve diagnostic practice [14]. The patients who have previously consented to the research use of their data and specimens have made progress possible. Some argue that new patients who benefit from these improvements have an ethical obligation to contribute themselves. Consequently, researchers should have access to hospital data for secondary research without active consent from patients [18]. Others argue that during national or international pandemic outbreaks it is vital to have the broadest samples possible [2, 19]. The COVID-19 pandemic exemplifies a clinical situation in which there is an urgent need for reliable data.

The results from empirical studies on patients’ opinions, like ours, should be kept in mind when discussing the balancing of autonomy and society’s interest in the greater good.

### Secondary research on genetic information

Regarding secondary research on genetic information, the patients had similarly positive attitudes. 81% of the respondents would allow it without active consent. Over 90% wanted to know if the genetic tests disclosed an increased risk of serious future illness. Even in cases where the illness could not be treated or prevented, more than half of the respondents still wanted the information. Hence the patients were much more interested in information about their health than in general information about researchers using their health information and biospecimens. These findings are in line with findings in a population of patients treated for cancer [6], indicating that a majority of patients are interested in information about their (future) health status if it is available.

### Data linkage, data sharing and confidentiality

The respondents also had positive attitudes regarding linkage of their hospital data to other sources of information. Only 1% of the patients refused, while another 9.4% wanted to be informed about potential linkages. Thus, 90% of the patients accept that health information from hospitalizations is linked to information in large national registries like the Cancer Registry, the Education Registry, and the Cause of Death Registry. This is in accordance with a study on lay people’s views on data linkage research [20].

Using a vast number of variables increases the possibility of reverse identification of single individuals, even though the data stems from sources that are deidentified. However, the results from the present study support a general impression that patients want research to be conducted. In our study the state of the data (i.e. identifiable vs. deidentified) was not specified in most of the questions. In other studies however the distinction between identifiable and anonymous data has previously been shown to be important for patients indicating the need for informed consent when there are risks of confidentiality or privacy loss [21]. There are also studies which indicate that patients prefer to know what they contribute to regardless of risks to privacy or confidentiality [22]. Patient opinions might vary between countries, influenced by cultural background and the population’s general trust in the health authorities.

Previous surveys have indicated that patients are reluctant to accept research on their biospecimens performed in foreign countries [2, 6]. The present study population had a different view. More than half of the respondents would accept that their biospecimens be shared “around the world” without receiving any information about this. Another 33% were satisfied with giving passive consent. Only 1% of the patients were negative towards passive consent. The same trends applied for health data. The lack of previous studies from hospital patient populations makes it difficult to interpret these differences. Again, we hypothesize that a populations’ general trust in health authorities might explain parts of the observed discrepancy. Future research addressing this topic would be informative since data sharing and confidentiality is becoming more and more relevant with an increased focus on international cooperation.

### Health data and the industry

In the present study, 95% of respondents were positive towards the use of their biospecimens in research involving business enterprises when the main aim was to provide better treatments. The number dropped to 33% when the main aim was economic profit. One might object that the questions were formulated without nuance. The respondents were forced to accept the premise that these aims can be separated, which is not necessarily true. However, it enabled us to unravel that the main aim of the research is decisive. Research for the common good is highly desirable, but research to profit the few is not. Warner et al. (2018) made similar findings [23]. Donors only “found it moderately acceptable that researchers at for-profit companies used the biospecimens” [6]. Reasons may be that the public, as well as the patients, is unaware of the important research conducted by commercial entities. Limited understanding of how research can translate into effective drugs suggest a need for public education in this area [6].

### Specific versus broad consent

Under certain conditions, a model with “broad consent” has support from patients, researchers, and ethicists [2, 6, 24]. This model gives donors or patients sufficient control over the use of their biospecimen, while the burdens and costs for researchers remain acceptable [2]. In the present study, the information accompanying the item regarding specific or broad consent was quite extensive (see Additional file 2). A majority of 62.5% preferred a broad consent model. However, the minority of 37.5% who preferred specific information is considerable. Generally, the present study population had a very positive attitude towards secondary research being conducted without active consent, but a substantial minority still wanted to be informed. Altogether, the findings in the present study are comparable to previous findings. For the majority of biospecimen donors, the concrete purpose of a study is less important [2]. However, there are also studies showing that members of the public are ambivalent about the use of broad consent for the use of biobank specimens due to concerns regarding certain types of research [25–28]. The present study may indicate that the purpose of a study is more important for hospital patients than for biospecimen donors from other contexts.

### Strengths and limitations

The present survey was conducted in a population of patients recently discharged from different hospital departments. The participants had been hospitalized between three and five months before they answered the questionnaire. They were included in a systematic manner. The relatively short interval between the hospital stays and the survey makes it probable that the themes addressed are relevant to the respondents. Their recent experiences as hospital patients may indicate an increased ability to personally reflect upon each item in the questionnaire. Previous surveys on the same subject have, to a large degree, been conducted in non-clinical or mixed samples. Thus, comparisons to other surveys must be made with caution.

Labeling, language, and conceptual clarity are important in surveys. Participants’ or patients’ responses are influenced by the overall presentation of the survey, as well as the formulation of single items or questions [14]. Biospecimens, for instance, can also be called “biological samples,” “specimens,” “human biological material,” or “donor material” [2]. The terms that are used influence how questions are perceived and complicate possible comparisons between studies. The undefined state of the hospital data and biospecimens is recognized as a limitation.

The response rate (RR) to the survey was 41%. This may be regarded as low and thus as a limitation.

On the other hand, the response representativeness in a study sample may be regarded as at least as important as the RR [29]. Looking at Table 1, only expected differences appeared. Cancer patients are known to more often participate in research as compared to other patients, and psychiatric patients less often. Likewise, older patients participate more often than younger patients. However, the respondents were all patients willing to respond in our questionnaire survey, and hence perhaps more willing to consent in the first place. This might present a bias. Beyond this, the population of respondents included patients from a wide range of hospital departments and severity of illnesses. Furthermore, all ages are represented and so are both sexes. Still, we are cautious to claim that the respondent group is representative for the population of newly discharged patients in general.

## Conclusions

The present study demonstrates that the respondent group was very positive to secondary research and support the concept of opting out rather than opting in. Keeping patients informed of planned research is also in line with the requirements of the General Data Protection Regulation (GDPR) and enables participants to exercise their rights, including the right to access information about oneself and the right to withdraw from participation. Our results also suggest that the model of broad consent is acceptable. Based on the voices of these patient representatives, we advocate for a liberalization of the consent policy in national legislations and hence the practices of IRBs/ECs/REBs [15, 30].

## Supplementary Information

**Additional file 1.** Information to participants. The information text sent to the participants

**Additional file 2.** Questionnaire. The questionnaire sent to all the participants

**Additional file 3.** Respondents’ answers to questionnaire. A summary of the respondents’ answers as frequencies and percentages for all twelve questions in the questionnaire

## Data Availability

The dataset collected and analyzed during the current study are available from the corresponding author on reasonable request.

## Abbreviations

EC: Ethical committee
GDPR: General data protection regulation
IRB: Institutional review board
REB: Research ethics boards
RR: Response rate
