# Supplementary material for: Research based on existing clinical data and biospecimens: a systematic study of patients’ opinions
Source: BMC Med Ethics. 2022 Jun 16;23:60. doi: 10.1186/s12910-022-00799-4 (PMC9202664; doi:10.1186/s12910-022-00799-4)
Supplement: Supplementary file 1 — Additional file 1. Information to participants. The information text sent to the participants [file 12910_2022_799_MOESM1_ESM.docx]

09.03.2018


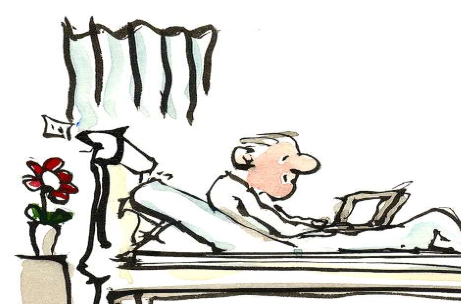
Would you be willing to participate in a survey on medical research?

WHY IS THIS STUDY BEING UNDERTAKEN?

The objective of this study is to obtain more knowledge about the attitudes to and opinions on medical research among former patients. Its aim is to use this knowledge in assessing different research projects that apply for ethical approval.

WHY ARE WE ASKING YOU IN PARTICULAR?

You are being asked to participate in this survey because you have previously been admitted to or attended an outpatient consultation at St. Olavs Hospital, Trondheim University Hospital. When you were a patient, health information (e.g. medical history, symptoms, test results) about you was collected. Test samples (blood, tissue, saliva, urine) may also have been collected from you during examination and treatment. Now that your examination or treatment is completed, your health information and test samples, if any, can be valuable for researchers. The researchers do not participate in your treatment as a patient, but wish to study the disease for which you were examined or treated, as well as other diseases. Studying your information and test samples may lead to new knowledge, and thereby to development of better examination and treatment, and new drugs.

WHAT WILL WE ASK YOU ABOUT?

The questions focus on what you think is okay or not okay when it comes to how the researchers use your health information and test samples after your stay in hospital.

WHO ARE WE?

We work in the Regional Committee for Medical and Health Research Ethics for Central Norway (REK). REK is charged with deciding what medical research projects are ethically appropriate to conduct on people. REK’s primary responsibility is to protect you as a research participant. To enable us to make the best possible decisions, it is very useful for us to know your opinion as a potential research participant.

We hope that you are willing to set aside approximately six minutes to answer these questions. You give consent by sending us the questionnaire – preferably as soon as possible. The answers you give will not be linked to your name, and participation is completely voluntary. To show our appreciation you will be entered into a prize draw for two iPad Air. For the prize draw, the researchers will randomly select two completed questionnaires to be sent to the hospital, which will then find the names and contact information of the winners so that they can be contacted.

Thank you in advance for taking the time to answer. Your contribution matters!

Your sincerely,

Hilde Eikemo Linda T. Roten Arne E. Vaaler

Head of the secretariat Advisor Professor, dr. med.

REK Central Norway REK Central Norway St. Olavs Hospital and REK Central Norway

09.03.2018

IF YOU DO NOT WANT TO PARTICIPATE

Irrespective of whether you want to participate in the survey or not, we have planned to register some information about you (gender, age group, hospital department). If you are opposed to such registration you can send the slip below (without signing it) in the enclosed reply-paid envelope within 14 days after your receipt of this letter.

# SLIP

SERIAL NUMBER: ________________
