# Supplementary material for: Research based on existing clinical data and biospecimens: a systematic study of patients’ opinions
Source: BMC Med Ethics. 2022 Jun 16;23:60. doi: 10.1186/s12910-022-00799-4 (PMC9202664; doi:10.1186/s12910-022-00799-4)
Supplement: Supplementary file 2 — Additional file 2. Questionnaire. The questionnaire sent to all the participants [file 12910_2022_799_MOESM2_ESM.docx]

|  |  |  |  |
| --- | --- | --- | --- |

**QUESTIONNAIRE**

Please choose only one response alternative for each question.

1. As a main rule in medical research, you as a potential research participant should be informed about the research project and asked to give consent (= confirm by your signature that you want to participate). In exceptional cases, the Regional Committee for Medical and Health Research Ethics (REK) may approve research projects without asking for your consent. Sometimes you may thus be informed about the project and that you may decline to participate (= you will be included in the research project unless you decline), other times you will receive no information whatsoever and remain unaware that you are participating in any research.

During your hospitalisation, various types of health information (e.g. medical history, symptoms, test results) were collected and registered in your patient records. Do you think it is okay for such health information to be used for research purposes later?

Yes, but I must be informed and give written consent to be included

Yes, and it will be enough to inform me that I’m included, unless I decline

Yes, and I need no information No

1. Occasionally, unused test samples (e.g. blood, tissue, saliva, urine) remain after examination and treatment in hospital. As a rule, these samples are discarded after a certain time. For researchers, however, such material is very valuable. Sometimes they therefore want to use it for research, rather than it being discarded. Do you think it is okay for such test samples that remain after your hospitalisation to be used for research purposes?

Yes, but I must be informed and give written consent to be included

Yes, and it will be enough to inform me that I’m included, unless I decline

Yes, and I need no information No

1. From your test samples, the researchers can produce information on your hereditary material/your genes. Your genes can provide a lot of information about you and your close relatives, e.g. indications of future illness. Do you think it is okay for research to be conducted on your genes?

Yes, but I must be informed and give written consent to be included

Yes, and it will be enough to inform me that I’m included, unless I decline

Yes, and I need no information

No

1. If researchers by using genetic research discover that you are at an increased risk of serious future illness, would you want to be informed of this?

Yes, if the disease can be treated or prevented

Yes, even if no treatment for the disease is available No

1. In Norway, we have for decades kept various registries (e.g. the Cancer Registry, the Education Registry, the Causes of Death Registry) that collect information of all residents. For the researchers, the health information from your hospitalisation will be even more valuable if it can be linked to information about you in the large registries. Do you think that making such a linkage is okay?

Yes, but I must be informed and give written consent to be included

Yes, and it will be enough to inform me that I’m included, unless I decline

Yes, and I need no information No

1. Do you think it is okay for researchers to use your test samples in collaboration with business enterprises (e.g. pharmaceutical companies) for research projects whose main aim is to provide better treatment to patients?

Yes

No

1. Do you think it is okay for researchers to use your test samples in collaboration with commercial operators/business partners (e.g. pharmaceutical companies) in research projects whose main aim is to earn a profit from the use of your test samples?

Yes

No

1. The Norwegian Institute of Public Health administers a registry called the Registry of Withdrawal of Biological Research Consent where you can register if you do not want test samples that have been collected in hospital to be used for research without your consent. Have you heard about the Registry of Withdrawal of Biological Research Consent before?

Yes

No

1. It has become fairly common for researchers to collaborate internationally to improve the reliability of their results. Do you think it is okay for your health information to be shared (without revealing your name) with other researchers all over the world?

Yes, but I must be informed and give written consent to be included

Yes, and it will be enough to inform me that I’m included, unless I decline

Yes, and I need no information No

1. Do you think it is okay for your test samples to be shared (without revealing your name) with other researchers all over the world?

Yes, but I must be informed and give written consent to be included

Yes, and it will be enough to inform me that I’m included, unless I decline

Yes, and I need no information No

1. Research distinguishes between specific consent and broad consent.

Specific consent means that the researchers ask you to participate in a specific project which is described in detail in an information letter to you. This means that you will receive a new letter for each new research project to be undertaken. Broad consent means that the researchers ask you to participate in many different projects that are not described in detail, but that all relate to the same topic (e.g. cancer). This means that you are asked only once in a single information letter.

Do you prefer to be asked for specific consent or broad consent?

I prefer specific consent

I prefer broad consent

1. If you could choose between giving consent electronically (by email/text message/online or in a regular letter by mail, what would you prefer?

I prefer to give consent electronically

I prefer to give consent on paper/in a letter

# FINALLY, WE ARE ASKING FOR SOME INFORMATION ABOUT YOU

Gender: Woman Man

Age: 18-29 30-39 40-49 50-59 60-69 70+

How serious do you consider your present illness to be?

Not serious at all

A little serious

Fairly serious Very serious

Is your condition wholly or partly due to genetic (hereditary) factors?

Not at all

Possibly

Most likely

Don’t know
