# Supplementary material for: Research based on existing clinical data and biospecimens: a systematic study of patients’ opinions
Source: BMC Med Ethics. 2022 Jun 16;23:60. doi: 10.1186/s12910-022-00799-4 (PMC9202664; doi:10.1186/s12910-022-00799-4)
Supplement: Supplementary file 3 — Additional file 3. Respondents’ answers to questionnaire. A summary of the respondents’ answers as frequencies and percentages for all twelve questions in the questionnaire [file 12910_2022_799_MOESM3_ESM.docx]

**Additional file 3:** Summary of the responders’ answers in frequency and percent.

|  | **Frequency** | **Valid percent** |
| --- | --- | --- |
| **Question 1** |  |  |
| Yes, but I must be informed and give written consent to be included | 50 | 11.8 |
| Yes, and it will be enough to inform me that I’m included, unless I decline | 179 | 42.3 |
| Yes, and I need no information | 193 | 45.6 |
| No | 1 | 0.2 |
| Total | 423 | 100.0 |
|  |  |  |
| **Question 2** |  |  |
| Yes, but I must be informed and give written consent to be included | 37 | 8.7 |
| Yes, and it will be enough to inform me that I’m included, unless I decline | 147 | 34.8 |
| Yes, and I need no information | 239 | 56.5 |
| No | 0 | 0.0 |
| Total | 423 | 100.0 |
|  |  |  |
| **Question 3** |  |  |
| Yes, but I must be informed and give written consent to be included | 72 | 17.0 |
| Yes, and it will be enough to inform me that I’m included, unless I decline | 182 | 43.0 |
| Yes, and I need no information | 160 | 37.8 |
| No | 9 | 2.1 |
| Total | 423 | 100.0 |
|  |  |  |
| **Question 4** |  |  |
| Yes, if the disease can be treated or prevented | 145 | 36.4 |
| Yes, even if no treatment for the disease is available | 218 | 54.8 |
| No | 35 | 8.8 |
| Total | 398 | 100.0 |
|  |  |  |
| **Question 5** |  |  |
| Yes, but I must be informed and give written consent to be included | 40 | 9.9 |
| Yes, and it will be enough to inform me that I’m included, unless I decline | 159 | 39.2 |
| Yes, and I need no information | 202 | 49.8 |
| No | 5 | 1.2 |
| Total | 406 | 100.0 |
|  |  |  |
| **Question 6** |  |  |
| Yes | 386 | 95.1 |
| No | 20 | 4.9 |
| Total | 406 | 100.0 |
|  |  |  |
| **Question 7** |  |  |
| Yes | 135 | 33.3 |
| No | 270 | 66.7 |
| Total | 405 | 100.0 |
|  |  |  |
| **Question 8** |  |  |
| Yes | 73 | 18.0 |
| No | 332 | 82.0 |
| Total | 405 | 100.0 |
|  |  |  |
| **Question 9** |  |  |
| Yes, but I must be informed and give written consent to be included | 40 | 9.6 |
| Yes, and it will be enough to inform me that I’m included, unless I decline | 159 | 38.2 |
| Yes, and I need no information | 202 | 48.6 |
| No | 5 | 1.2 |
| Total | 416 | 100.0 |
|  |  |  |
| **Question 10** |  |  |
| Yes, but I must be informed and give written consent to be included | 44 | 10.6 |
| Yes, and it will be enough to inform me that I’m included, unless I decline | 135 | 32.5 |
| Yes, and I need no information | 228 | 54.9 |
| No | 8 | 1.9 |
| Total | 415 | 100.0 |
|  |  |  |
| **Question 11** |  |  |
| I prefer specific consent | 156 | 37.5 |
| I prefer broad consent | 260 | 62.5 |
| Total | 416 | 100.0 |
|  |  |  |
| **Question 12** |  |  |
| I prefer to give consent electronically | 240 | 57.7 |
| I prefer to give consent on paper/in a letter | 176 | 42.3 |
| Total | 416 | 100.0 |
|  |  |  |
